# Pathogenesis of Dengue: Dawn of a New Era

**DOI:** 10.12688/f1000research.7024.1

**Published:** 2015-11-25

**Authors:** Scott B. Halstead

**Affiliations:** 1 Dengue Vaccine Initiative, International Vaccine Institute, Seoul, Korea, South

**Keywords:** Dengue virus, hemorrhagic fever, dengue shock syndrome, dengue vascular permeability syndrome, Dengue pathogenesis, viral toxicosis

## Abstract

Dengue virus (DENV) infections of humans were long thought to be self-limited and of low mortality. Beginning in the 1950s, at the time when four different DENVs were discovered, a lethal variant of dengue emerged. Dengue hemorrhagic fever/dengue shock syndrome (DHF/DSS) initially observed in Southeast Asia now has spread throughout the world. Two risk factors for DHF/DSS are well-established: severe disease occurs during a second heterotypic DENV infection or during a first DENV infection in infants born to dengue-immune mothers. A large number of hypotheses have been proposed to explain severe dengue disease. As discussed, few of them attempt to explain why severe disease occurs under the two different immunological settings. New experimental evidence has demonstrated that DENV non-structural protein 1 (NS1) is toll-receptor 4 agonist that stimulates primary human myeloid cells to produce the same cytokines observed during the course of severe dengue disease. In addition, NS1 directly damages endothelial cells. These observations have been repeated and extended to an in vivo mouse model. The well-established phenomenon, antibody-dependent enhancement of DENV infection in Fc-receptor-bearing cells, should similarly enhance the production of DENV NS1 in humans, providing a unitary mechanism for severe disease in both immunological settings

## Introduction

During the first half of the 20th century, human responses to dengue virus (DENV) infection were described in multiple studies on hundreds of adult volunteers in different parts of the world ^1– 9^. On the basis of these case descriptions, many earlier 19th and 20th century outbreaks were identified as dengue fever (DF). This large historical experience failed to prepare the scientific community for a surprise in 1956. DENVs were identified as the cause of a fatal hemorrhagic fever in Southeast Asian children with few features of DF ^10, 11^. Ever since, the question has been “why did dengue turn deadly?”

In the 1960s, dengue research programs in Southeast Asia found that children were dying of a new clinical syndrome, an acute febrile disease accompanied by a complex of physiologic abnormalities affecting multiple organ systems including the liver, blood coagulation, complement, hematopoiesis, serum proteins, and the vascular system that reach maximal expression at defervescence. Initially, this entity was named the dengue shock syndrome (DSS) and sub-shock, dengue hemorrhagic fever (DHF) ^12– 16^. However, the disease described in some detail in Box 1 is best identified as the dengue vascular permeability syndrome (DVPS).

**Box 1. DVPS Clinical Presentation**

There is an abrupt onset of fever accompanied by malaise, vomiting, headache, anorexia, abdominal pain and upper respiratory symptoms. Two to five days later the patient may rapidly deteriorate and collapse. At or near defervescence, the patient may have cold, clammy extremities, slow venous filling, a warm trunk, flushed face, circumoral and peripheral cyanosis, diaphoresis, restlessness, irritability, mid-epigastric pain, decreased urinary output and hypovolemia. There may be scattered petechiae on the forehead and extremities or spontaneous ecchymoses, bruising and bleeding at sites of venipuncture. Respirations are rapid. The pulse is weak, rapid, and thready and the heart sounds are faint. The liver may enlarge to 4–6 cm below the costal margin and is usually firm and somewhat tender. Laboratory findings during acute stage illness include thrombocytopenia, elevated liver enzymes, activated complement with high levels of C3a and C5a, fibrin split products, low fibrinogen, prolonged bleeding time, prolonged APTT, low serum albumin and elevated hematocrit ^13, 17, 85, 86^.

A critical loss of fluid and smaller macromolecules through damaged endothelium may result in reduced blood volume and an increase in hematocrit. A hematocrit that is 20% or greater than a convalescent value denotes cardiovascular instability. Vascular leakage can be detected directly by x-ray or sonography. Pleural effusions are best detected by lateral view chest x-ray. Abdominal sonograms may detect gall bladder wall thickening and serosal effusions ^87^. Approximately 20–30% of cases of DVPS develop shock with an onset that can be subtle, arising in patients who are fully alert, and accompanied by increased peripheral vascular resistance. Shock is not due to congestive heart failure but to hypovolemia. With increasing cardiovascular compromise, diastolic pressure rises towards systolic and pulse pressure narrows to less than 20 mm Hg. Fewer than 10% of patients have gross ecchymosis or gastrointestinal bleeding, usually after a period of uncorrected shock.

After a 24- to 36-hr period of crisis, the vascular leak self-heals and convalescence is fairly rapid and complete. Encephalopathy may be seen during this period. Rare complications are myocarditis and hepatitis. Bradycardia and ventricular extrasystoles are common during convalescence ^87^.

Etiological studies discovered that hospitalized DVPS occurs in two immunological settings: 1) approximately 90% with secondary-type DENV antibody responses were shown epidemiologically to accompany a second heterotypic DENV infection, and 2) approximately 5% of cases were infants born to dengue-immune mothers who had primary DENV antibody responses ^17– 19^. Important caveats have been discovered for each of these two immunological risk factors. Hospitalized DVPS accompanying a second heterotypic dengue infection is a rare event. Approximately 2% to 4% of secondary dengue infections resulted in hospitalized DHF/DSS ^20– 23^. Young children are inherently at greater risk of developing DVPS during a second heterotypic dengue infection than are older children or adults ^24^. When sequential DENV infections are closely spaced, there is significant cross-protection ^25^. This cross-protection initially prevents infection by a second DENV but it persists in partial form, preventing DVPS as a component of the disease response for at least two years ^26, 27^. As the interval between sequential DENV infections increases beyond two years, an ever larger fraction of second heterotypic DENV 2 and 3 infections have culminated in severe DVPS ^28, 29^. DVPS in infants usually occurs during the second half of the first year of life. Passive antibodies from mothers, known to have been infected by multiple DENV earlier in life, protect against DENV infections for a period of months, then mediate DVPS and finally disappear at around 12 months ^18, 30^. DVPS occurs more frequently during DENV infections of infants with passively acquired DENV antibodies than in older children accompanying a second DENV infection ^30, 31^.

In the decades after DHF and DSS were first described, numerous observations have been made in dengue-infected individuals of all ages, and hypotheses have been put forward to explain the mechanism of severe and fatal dengue. Roughly in chronological order, those attracting the most attention are the following:

### 1. Antigen-antibody-complement-mediated vascular permeability

During a second heterotypic dengue infection, the simultaneous circulation of anamnestic IgG dengue antibodies and dengue viral antigens activates complement via the C3 activator and by initiating the C1, C4, C2 cascade, contributing to a reduced level of C3. The resulting increased levels of C3a and C5a anaphylatoxins are thought to mediate vascular permeability ^14, 15, 32^.

### 2. Antibody-dependent enhancement of dengue infection

This phenomenon was discovered when it was observed that DENV readily grew *in vitro* in cultures of peripheral blood monocytes obtained from dengue-immune monkeys or humans but less well in monocytes from non-immunes ^33, 34^. Soon after, it was discovered that this phenomenon was readily mediated by dengue antibodies that were diluted above the neutralization endpoint, added to dengue viruses, and grown in cultured monocytes from seronegative donors ^35, 36^. In rhesus monkeys, enhanced viremias were observed *in vivo* during secondary compared with primary DENV 2 infections ^37^. Enhanced DENV 2 viremias were also produced in susceptible monkeys sensitized with a small intravenous dose of dengue-immune human cord blood serum ^38^.

### 3. Exaggerated T-cell response

Vascular permeability has been attributed to cytokines, such as interleukin-2 (IL-2) and tumor necrosis factor-alpha (TNFα), released by cohorts of overactive T cells that accompany immune responses during a second heterotypic DENV infection ^39, 40^.

### 4. Virulent dengue viruses

The concept of “virulent” or “non-virulent” dengue viruses developed when it was observed that the American genotype of DENV 2 did not produce a large outbreak of DHF/DSS in Iquitos, Peru, during a 1995 outbreak in a population that was highly immune to DENV 1 ^41, 42^. Also, pronounced differences in clinical expression of infections caused by a genotype of DENV 2 were observed in outbreaks on different Pacific islands ^43^.

### 5. Heterophile immunity

In the extensive experimental literature describing observations in mouse models, it is proposed that DVPS is a short-lived autoimmune disease resulting from destructive tissue responses to pathogenic antibodies raised to DENV NS1 proteins. These antibodies cross-react with host endothelial cells, blood-clotting proteins, and liver cells. Mimetic antibodies are thought to reach pathological levels during secondary DENV infections ^44– 46^.

### 6. Infection-ending T-cell responses misdirected by original antigenic sin

Analysis of the functional phenotypes of CD8 ^+^ T cells in DHF cases revealed that recognition between different DENV peptides was associated with reduced cytolytic potential without reducing cytokine production ^47– 49^. Activation of both CD4 ^+^ and CD8 ^+^ T cells with peptide variants induced different sets of cytokines ^50^. Pathogenic heterologous T-cell responses or selectively defective T-cell responses (original antigenic sin) result in cytokines and chemokines (“cytokine storm”) that produce vascular permeability leading to DHF/DSS. T-cell responses enhance the severity of DENV infections by an *in vitro* process that has not been demonstrated *in vivo* ^47, 51– 53^.

### 7. Direct infection of myeloid cells

A host of *in vitro* studies suggest that vascular permeability results from cytokines or other factors generated by DENV infection of myeloid cells, including mast cells ^54– 59^.

### 8. Direct infection of endothelial cells

Dengue viruses readily grow in primary human endothelial cell explants, generating products that increase vascular permeability ^60^. Transcriptional activity, protein production, and cell surface protein expression by endothelial cells are significantly altered by DENV infection *in vitro*. Several pathways identified in DHF/DSS, including inflammation, apoptosis, and coagulation, are affected ^61, 62^. Apoptosis of endothelial cells has been demonstrated in mice and has been proposed to be the mechanism of vascular leakage ^63^.

## Which is the more relevant pathogenic mechanism?

Only one of these hypotheses satisfies the requirement of “Occam’s razor” (among competing hypotheses, the one with the fewest assumptions should be selected), providing a hypothesis that offers the simplest explanation why DVPS occurs in persons who are actively or passively dengue-immune. One hypothesis, antibody-dependent enhancement of dengue infection (ADE) (#2), satisfies this requirement, whereas hypotheses #1 (acute immune complex disease), #3 (exaggerated T-cell response), #5 (heterophile immunity), and #6 (original antigenic sin) do not. These hypotheses are unable to explain why DVPS accompanies a primary DENV infection in infants who circulate passively acquired dengue antibodies. Hypothesis #4 (virulent DENV) suggests that it is the innate properties of different genotypes or strains of dengue viruses that control the outcome of disease. DVPS, carefully documented, has not accompanied DENV infections in naïve populations. Dengue viral contributions to DVPS are conditioned by pre-illness dengue antibodies. This is not to say that differences between DENV strains or genotypes may not interact with antibodies to profoundly change biological outcomes. This important possibility is partially discussed at greater length elsewhere ^64, 65^. This pathogenesis-relevant antibody-dependent phenomenon is best described as “fitness” and not as “virulence”. Hypothesis #7, when confined to experimental studies on the implications of direct infection, predicts that any DENV infection, regardless of immunological status, may result in DVPS. Hypothesis #8 attributes DVPS to the direct viral infection of endothelial cells. The problem here is the paucity of high-quality studies. The reagents needed to identify sites of DENV replication in human autopsy tissues are scarce, present difficult quality-control challenges, and are not standardized throughout the dengue research community. In studies using anti-NS3 staining, DENV antigens have been localized to focal endothelial cells in several organs. But the distribution and intensity of staining is unlike that observed with the direct infection of endothelial cells in hantaviral pulmonary syndrome associated with severe localized vascular permeability ^66– 68^. A recent effort made to identify endothelial cells stained with DENV antigens was negative ^69^.

## Dengue pathogenesis: ADE and dengue viral toxicosis

All infectious diseases are kinetic, consisting of the invasion of the microorganism (afferent phase) followed by the host response (efferent phase), including disease and elimination of the organism. The setting of DENV infections is unique in that pre-infection events may control afferent phenomena. During the afferent phase of dengue infections, DENV infectious immune complexes regulate infection of Fc-receptor-bearing cells. Antibodies, whether passively or actively acquired, on some, but not all occasions, intervene with infection dynamics to produce an expanded infected cell mass. As predicted by experimental ADE studies, early acute-phase illness sera from children with second DENV infections were found to have higher peak viremia and antigenemia titers prior to the onset of DHF/DSS than did similarly timed sera from children who developed a milder illness ^70– 72^. More recently, Vietnamese and Sri Lankan researchers observed the circulation of NS1 at higher titers and longer intervals during severe disease ^73, 74^. Afferent ADE has been successfully modeled *in vivo*. DENV 2, 3, and 4 infections in type I and II interferon receptor-deficient mice produce a non-paralytic lethal disease accompanied by many features of DVPS, high levels of virus in tissues and circulating in blood and efferent phenomena, a cytokine storm, low platelet counts, elevated hematocrit, increased vascular permeability, and intestinal hemorrhage ^75– 77^. Mice transfused with enhancing concentrations of dengue antibodies prior to infection with a sub-lethal dose of mouse-adapted DENV 2 developed lethal vascular permeability with TNF release ^78, 79^. It is now known that dengue immune complex infection of human monocytes/macrophages boosts DENV replication approximately 100-fold in association with the suppression of type I interferon or increased production of IL-10 or both. This phenomenon is called intrinsic ADE (iADE) ^80, 81^. Infectious immune complexes also achieve a threefold infection advantage in FcR-bearing cells compared with DENV alone (extrinsic ADE– eADE) ^82^.

What causes the efferent signs and symptoms of DVPS? Missing is the “smoking gun” that produces liver injury, vascular permeability, activation of complement, and alteration of hemostasis. Very recently, Paul Young’s group observed an analogy between the cellular biology of bacterial lipopolysaccharides (LPS) and that of DENV NS1 ^83^. Each of these compounds interacts with Toll-like receptor 4 (TLR 4) on the surface of monocytes, macrophages, and endothelial cells, inducing the release of a range of cytokines and chemokines. These are the same mediators identified in the blood of patients with DHF/DSS. *In vitro*, NS1 resulted in the disruption of endothelial cell monolayer integrity. The authors conclude that DSS may be a viral protein toxicosis. NS1-mediated cytokine release was inhibited by the TLR4 antagonist LPS- *Rhodobacter sphaeroides*, suggesting an avenue for therapeutic intervention. Crucially, this same observation has been confirmed in an *in vivo* model. The Harris laboratory has shown that DENV 2 NS1 inoculated intravenously at physiologically relevant concentrations in sub-lethal DENV 2-infected IFNAR ^−/−^C57BL/6 mice produced lethal vascular permeability ^84^. *In vitro*, NS1, when added to cultured endothelial cells, resulted in endothelial permeability. Vaccination of mice with DENV 2 NS1 protected against endothelial leakage and death due to lethal DENV 2 challenge. Mice immunized with DENV 2 NS1 protein were completely protected against homologous DENV 2 challenge, and immunization with DENV 1, 3, and 4 NS1 proteins partially protected against heterologous DENV 2 challenge.

DENV NS1 blood levels and therefore NS1 toxicosis are efferent mechanisms directly controlled by ADE. It is not clear yet whether NS1 alone, NS1-induced cytokines, virus replication-induced damage, or activated complement is responsible for the *in vivo* efferent DVPS phenomenon. There is a delay of several days between the early occurrence of peak blood levels of NS1 and defervescence-associated organ pathology. This is not fully understood. It is clear, however, that DENV NS1 toxicosis introduces a new era to dengue pathogenesis research. 
